# Feasibility of Virtual Reality Exercises at Home for Post–COVID-19 Condition: Cohort Study

**DOI:** 10.2196/36836

**Published:** 2022-08-15

**Authors:** Tjitske Groenveld, Retze Achttien, Merlijn Smits, Marjan de Vries, Ron van Heerde, Bart Staal, Harry van Goor, COVID Rehab Group

**Affiliations:** 1 Department of Surgery Radboud Institute for Health Sciences Radboud University Medical Center Nijmegen Netherlands; 2 Research Group Musculoskeletal Rehabilitation Hogeschool Arnhem en Nijmegen University of Applied Science Nijmegen Netherlands; 3 Scientific Center for Quality of Healthcare Radboud University Medical Center Nijmegen Netherlands; 4 See Acknowledgments

**Keywords:** virtual reality, rehabilitation, COVID-19, long COVID, feasibility, physical function, quality of life, pandemic, multimodal virtual reality, outpatient care, physiotherapy, digital health, patient care

## Abstract

**Background:**

Between 30% to 76% of COVID-19 patients have persistent physical and mental symptoms, sometimes up to 9 months after acute COVID-19. Current rehabilitation is mostly focused on the physical symptoms, whereas experts have agreed on the need for a biopsychosocial approach. A novel approach such as virtual reality (VR) rehabilitation at home might benefit patients and therapists, especially considering the expected rush of patients with post–COVID-19 condition needing rehabilitation.

**Objective:**

The aim of this study was to investigate the feasibility of self-administered VR exercises at home for post–COVID-19 condition.

**Methods:**

This was a single-arm feasibility study in an outpatient care setting. Patients who needed physiotherapy because of post–COVID-19 condition were included as determined by the treating physiotherapist. Participants performed VR physical exercises at home for a period of 6 weeks and were allowed to perform VR mental exercise through applications available on the VR platform to reduce stress and anxiety and promote cognitive functioning. The main outcomes were related to feasibility (ie, duration and frequency of VR use), safety (ie, adverse events), patient satisfaction, and reasons to withdraw. Physical performance, daily activities, cognitive functioning, anxiety and depression, and the quality of life were measured before and after.

**Results:**

In total, 48 patients were included; 1 (2%) patient did not start VR, and 7 (15%) patients withdrew, mostly due to dizziness. Almost 70% (33/47) of participants reported experiencing any adverse event during VR exercising. However, only 25% (9/36) recalled these events at the end of the intervention period. The majority (27/36, 75%) of the patients described VR as having a positive influence on their recovery, and the global satisfaction score was 67%. The average VR use was 30 minutes per session, 3-4 times a week for 3-6 weeks. The overall use of VR applications was almost equally distributed over the 3 sets of VR exercises (physical, relaxing, and cognitive). However, the use frequency of physical exercises seemed to decrease over time, whereas the use of cognitive and relaxation exercises remained stable. Physical performance and quality of life outcomes were significantly improved after 6 weeks.

**Conclusions:**

VR physical exercises at home is feasible and safe with good acceptance in a significant percentage of patient with post–COVID-19 condition.

**Trial Registration:**

ClinicalTrials.gov NCT04505761; https://clinicaltrials.gov/ct2/show/NCT04505761

## Introduction

The ongoing COVID-19 pandemic is leading to serious morbidity and mortality worldwide [1,2]. Studies show that 30% to 76% of COVID-19 patients have persistent symptoms, sometimes up to 9 months after acute COVID-19 [3-5]. These patients have a variety of symptoms in the physical and mental domains [6,7]. A substantial amount of post-COVID-19 patients experience limitations in daily activities and social participation in the long term [8,9]. This condition was described as “long COVID,” “Post-(acute-)COVID syndrome,” or “postacute sequelae of SARS-CoV-2 infection” and is now termed “post–COVID-19 condition” [10-12]. Patient-tailored post–COVID-19 rehabilitation is needed to recover these physical and mental functions and ultimately improve the patients’ quality of life [13,14].

Several reviews, consensus statements, and position papers concerning post–COVID-19 rehabilitation have already been put forth by professional rehabilitation organizations [13,15-18]. These experts agree on the need for an individualized program with a multimodal approach, not only aiming at restoring physical functioning, but also at reducing anxiety and depression and offering cognitive rehabilitation when needed. Virtual reality (VR) applications may be important tools in such rehabilitation, since they have the potential to address all aspects of this multimodal approach in a single solution [19]. Furthermore, they can provide health care practitioners with an easy-to-administer, tailor-made home rehabilitation solution against an impending surge of demand for post–COVID-19 rehabilitation.

VR has the ability to immerse someone into another world, which can be used to distract patients from experiencing pain, fatigue, and anxiety and may increase therapy adherence. VR is increasingly used in rehabilitation such as poststroke rehabilitation, limb rehabilitation, and the treatment of posttraumatic stress disorder [20-22]. The use of VR for the improvement of general physical condition and health is relatively new and often involves 2D “exergaming” [23,24]. Recently, VR relaxation games were used for inpatient post–COVID-19 rehabilitation, showing high patient satisfaction and benefits regarding stress reduction and cognitive functioning [25]. VR exercises for patients with post–COVID-19 condition outside of the hospital may have similar benefits. These exercises would enlarge access to rehabilitation resources in general and, more specifically, for the large group of patients with acute COVID-19 at home.

This study aimed to assess feasibility—usability, acceptability, tolerability, and safety—of 6 weeks of VR exercises at home indicated by community-based physiotherapists. Secondarily, we analyzed the changes in physical and mental functions and the quality of life.

## Methods

### Design

This was a single-arm study to primarily assess the feasibility regarding acceptability, usability, and tolerability and, additionally, the changes in physical and mental functions and the quality of life of 6 weeks of VR exercises at home. As part of this study, a digital health design evaluation was performed, which was separately reported [26].

### Ethics Approval

Ethical approval was obtained by the Research Ethics Committee of the Radboud University Medical Centre (2020-6770). The study was conducted according to the principles of the Helsinki Declaration and in accordance with Dutch guidelines, regulations, and acts (Medical Research involving Human Subjects Act) and registered at ClinicalTrials.gov (NCT04505761).

### Participants and Study Setting

The study population comprised of patients with post–COVID-19 condition referred for physiotherapy to a physiotherapist in a community-based practice or outpatient rehabilitation clinic in the southeast of the Netherlands between July 2020 and February 2021. The selection criteria are listed in Textbox 1. Proven COVID-19 by laboratory test was not an inclusion criterion, because a considerable number of patients with the acute disease at home were not tested in the study period. We considered an estimated duration of 3 weeks of physiotherapy as the minimum to properly investigate feasibility outcome parameters and avoid including patients with minimal or single symptoms. No sample size calculation was performed. Patients who withdrew from the study within 3 weeks were replaced to achieve a total number of 40 evaluable patients for the physical and mental functions and quality of life outcomes. Written informed consent was obtained from each participating patient. Participating physiotherapists were experienced in treating similar conditions such as Q fever and post-intensive care syndrome, and half (7/15, 47%) had completed a master’s degree in psychosomatic physiotherapy.

**Inclusion and exclusion criteria of patients with post–COVID-19 condition.**

**Inclusion criteria**

- Symptoms attributable to post–COVID-19 condition
- Indication for physiotherapy for rehabilitation after COVID-19
- Considered suitable for virtual reality home exercises by the treating physiotherapist
- Estimated duration of physiotherapy of at least 3 weeks
- Aged ≥16 years on inclusion date
- Willing and able to comply with the study protocol
- Read and speak Dutch

**Exclusion criteria**

- Patient participates in another study that interferes with this study
- One or more “red flags” for exercise in patients with COVID-19 (see Multimedia Appendix 1 [27])
- Severe anxiety or depression complaints
- High risk of contamination with therapy resistant microorganism, such as methicillin-resistant *Staphylococcus aureus*
- Patient has difficulties handling virtual reality in the following ways:
  Experiencing delirium or acute confusional state
  (A history of) dementia, seizure, or epilepsy
  Severe hearing or visual impairment that is not corrected
  The skin of the head or face is not intact (eg, head wounds, psoriasis, and eczema)

### Intervention

The intervention consisted of 6 weeks of VR physical exercises at home with the choice by the participants to additionally perform VR mental exercises. To this end, a VR suite was composed of off-the-shelf and custom-made applications for physical (SyncVR Fit; SyncVR Medical), cognitive (Koji’s Quest; NeuroReality), and relaxation exercises (SyncVR Relax & Distract; SyncVR Medical) in collaboration with SyncVR Medical and NeuroReality. SyncVR Fit comprises several game applications, such as goalkeeping or beach squats, each with a duration of approximately 10 minutes and 3 levels in difficulty. Koji’s Quest immerses players in a virtual environment designed to engage players through increasingly more challenging brain training activities combined with a reward system that encourages daily play. SyncVR Relax & Distract offers relaxation through games, videos, and meditation. Patients received written instructions on all applications (Multimedia Appendix 2). For the VR at home exercises, all patients were loaned an Oculus Quest head-mounted display (Facebook Technologies), which can bring the user into an immersive, realistic, and multisensory environment by computer-generated visuals. Participating physiotherapists were instructed on prescribing VR use by the research team. Prescription followed the guidelines of the Royal Dutch Society for Physiotherapy for post–COVID-19 physiotherapy with the type, time, and frequency of the exercises translated into VR exercises [27]. Prescription was individualized to the needs and impairments of the patient and as determined by baseline performance tests. However, patients were instructed not to exceed 30 minutes per session to avoid “VR sickness” [28]. Per the protocol, the prescription was meant to be adapted at follow-up contacts according to the patient’s feedback and digital VR tracking data. Due to organizational challenges and the practicality of conducting the study during the pandemic, this protocol was changed to instructing patients to choose the frequency and duration of VR use according to their preferences and needs. For safety reasons, the first few VR sessions were supervised by the physiotherapist in the office. When deemed safe, patients continued the VR exercises at home. Telerehabilitation, including remote monitoring and video consulting, was not part of the study procedure due to the workload of the physiotherapists and limited resources.

### Procedure and Measurements

After informed consent, patient and disease characteristics were documented. Physical performance metrics were administered by the physiotherapists as part of usual care and collected at the start and end of the intervention period of 6 weeks. Questionnaires were completed by the patients at the same time. Postintervention questionnaires were not administered to patients who withdrew from the study within 3 weeks. During the intervention period, patients were asked to keep a diary on the frequency and duration of their VR use, which applications they used, and if they experienced any adverse events. Weekly, short, and semistructured telephone interviews were carried out to monitor adherence and solve any (technical) problems. Furthermore, a 24/7 support line was available for questions or (technical) problems, and patients were encouraged to contact it when needed.

### Patient Characteristics

Patient characteristics included age, gender, the duration of symptoms, and prior hospital and intensive care unit admission for COVID-19. The duration of symptoms was defined as the number of months between the first day of COVID-19 symptoms and the first day of the VR exercises. Previous experience with digital technology was assessed, including smartphone, laptop, exergaming, smart home devices, and VR or augmented reality games. The Mentality test (Motivaction) was used to gain insight into the individuals’ opinions, motivation, and behavior toward (support in) health care and susceptibility for technology [29]. The Mentality test is a questionnaire consisting of 59 items. Based on the answers, patients are categorized as “less self-sufficient,” “pragmatic,” or “socially critical.”

### Outcome Measures

Primary outcome was feasibility, as reflected by end points regarding the acceptability, usability, tolerability, and safety of VR exercises. Secondary outcomes were physical and mental functions and the quality of life.

#### Acceptability

The discontinuation of the VR exercises was noted as an acceptability outcome, together with the reasons for withdrawal. Patient satisfaction was measured at the end of the intervention period by the Treatment Satisfaction Questionnaire for Medication [30], modified by replacing “medication” with “intervention.” Subscores were calculated for effectiveness, side effects, convenience, and global satisfaction, where 0 is extremely dissatisfied and 100 is extremely satisfied [30]. Furthermore, 2 questions were added: “If you would end up in the same situation in the future, would you want to use Virtual Reality again?” and “Would you recommend Virtual Reality to a friend or family member?”

#### Usability

The frequency and duration of VR use were assessed using the digital tracking feature that is incorporated in the VR intervention and a patient diary. Patients also noted technical difficulties affecting usability in the diary. The third source of usability data was the weekly semistructured telephone call and patient calls to study staff.

#### Tolerability and Safety

Tolerability was determined by registering adverse events in the diary through open-ended questions and the subscore “side effects” of the treatment satisfaction questionnaire. Additionally, for participants who withdrew from the study, any possible adverse events were registered. Safety was assessed by registering serious adverse events such as falls or near falls as reported by participants in the diaries or weekly telephone calls.

#### Physical Function

The 6-Minute Walk Test (6-MWT) was used to measure the overall physical condition of the participants [31]. When participants were not able to perform the 6-MWT, the Timed Up and Go Test (TUG) was performed [32]. Grip strength was used as an indicator of general strength [33]. The strength of the lower extremity was determined with the 30-Second Chair to Stand Test (30-CST) [34]. When the participant was not able to perform the 30-CST, the 5-times stand test (measured in seconds) was performed. Fatigue was assessed using an 11-point Borg scale (0=no fatigue and 10=maximal fatigue) [35]. The Patient-Specific Complaints (PSC) questionnaire was used to score the patients’ ability to perform 3 self-chosen daily activities [36]. The Nottingham Extended Activities of Daily Living (NEADL) score was used to measure to what degree a patient can independently perform the activities of daily living [37].

#### Mental Function and Quality of Life

The Hospital Anxiety and Depression Score (HADS) was used as a global measure of psychological distress. The cutoff for possible anxiety or depression disorder is 8 points [38]. The Short Form-12 (SF-12) was used to measure the health-related quality of life. Norm-based scores were calculated using the method described by Ware et al [39]. The Positive Health questionnaire was used to measure patients’ feelings about their different dimensions of health: overall health, bodily functions, mental well-being, meaningfulness, quality of life, participation, and daily functioning [40]. The higher the score, the better a patient feels about his or her health. The Cognitive Failure Questionnaire (CFQ) was used to measure subjective cognitive function [41].

### Data Analysis

All patients who started VR were included in the feasibility analyses. The VR applications used, as noted in the patient diaries, were categorized as physical, cognitive, or relaxing. To analyze the frequency and duration of VR use per week, patients were included when they reported having used VR at least once in the corresponding week. For analysis of (serious) adverse events, the free-text reports of participants were matched to the terminology of the Common Terminology Criteria for Adverse Events (version 5.0) and the definition of the US Food and Drug Administration for serious adverse events [42].

Only patients who used the VR exercises for 3 weeks or more and with baseline and final measurements were included in the analyses of physical and mental functions and quality of life metrics and questionnaires.

### Statistical Analysis

SPSS statistical software (version 25; IBM Corp) was used for statistical analyses. Descriptive statistics were used to analyze the outcomes of usability, acceptability, tolerability, safety, physical and mental functions, and quality of life. Dependent on the distribution of the data, paired samples 2-tailed *t* test or Wilcoxon signed-rank test was used to determine changes in the functions and quality of life of post–COVID-19 VR exercises. To evaluate which patients benefitted the most from VR, we explored correlations matrices and calculated Pearson correlation coefficient for the different combinations of patient and disease characteristics, duration of VR use, and physical and mental functions and quality of life outcomes. Post hoc subgroup analyses were performed regarding the use of cognitive and relaxation exercise applications (yes/no) and their respective outcomes on the CFQ and HADS.

## Results

### Patient Characteristics

**Figure 1:**
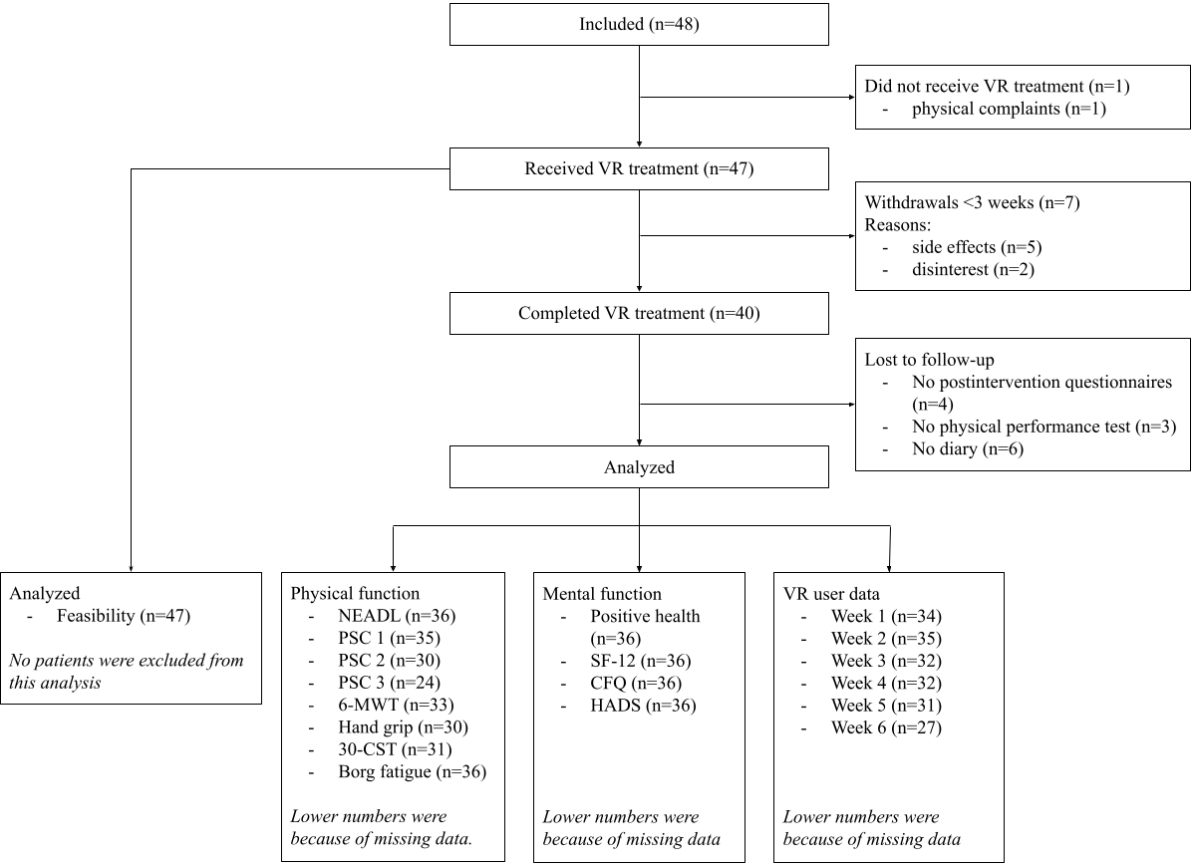
Study flowchart. 30-CST: 30-Second Chair to Stand Test; 6-MWT: 6-Minute Walk Test; CFQ: Cognitive Failure Questionnaire; HADS: Hospital Anxiety and Depression Score; NEADL: Nottingham Extended Activities of Daily Living score; PSC: Patient-Specific Complaints questionnaire; SF-12: Short Form-12; VR: virtual reality.

Between July 2020 and February 2021, 48 patients were included from 13 community-based physiotherapy practices and 1 rehabilitation clinic. In 66% (31/47) of the patients, COVID-19 was confirmed by a positive polymerase chain reaction test, and the remaining 34% (16/47) had signs and symptoms corresponding with COVID-19. The median age was 54 years, and 68% (32/47) was female (Table 1). There was 1 patient who experienced an acute onset of back pain before receiving VR treatment; the remaining 47 patients were eligible for the feasibility analyses (Figure 1).

**Table 1:** Patient and disease characteristics of 47 patients who were evaluated for feasibility.

Characteristic		Patient (N=47)
Age (years), median (IQR; range)		54 (39-59; 21-70)
Gender, female, n (%)		32 (68)
Duration of COVID-19 symptoms (months), median (IQR; range)		7.2 (4.3-8.2; 1.2-10.1)
**Hospital admission**		
	Patient admitted to hospital, n (%)	9 (19)
	Duration of hospital admission (days), mean (range)	21 (3-114)
	Missing, n (%)	4 (9)
**Intensive care unit admission**		
	Patient admitted to hospital, n (%)	5 (11)
	Duration of intensive care unit admission (days), mean (range)	10 (9-84)
	Missing, n (%)	4 (9)
**Mentality test, n (%)**		
	Less self-sufficient	11 (23)
	Pragmatic	22 (47)
	Socially critical	7 (15)
	Missing	7 (15)
Daily experience with ≥3 digital technologies^a^, n (%)		41 (86)
Previous experience with virtual reality, n (%)		12 (26)

^a^For example, smartphone, tablet, laptop, internet, and television.

### Outcomes

#### Acceptability

In total, 7 patients withdrew from the study within the first 3 weeks, 5 due to adverse events (dizziness, migraine, and blurred vision) and 2 because of lost interest. These patients were replaced according to the study protocol. Between weeks 3 and 5 in the study period, another 5 patients discontinued the VR exercises due to neck pain, dizziness, emotional processing of the post–COVID-19 condition, lost interest, and study logistics. Patients who lost interest mentioned that they found the games boring or had doubts about the value of the VR exercises.

**Figure 2:**
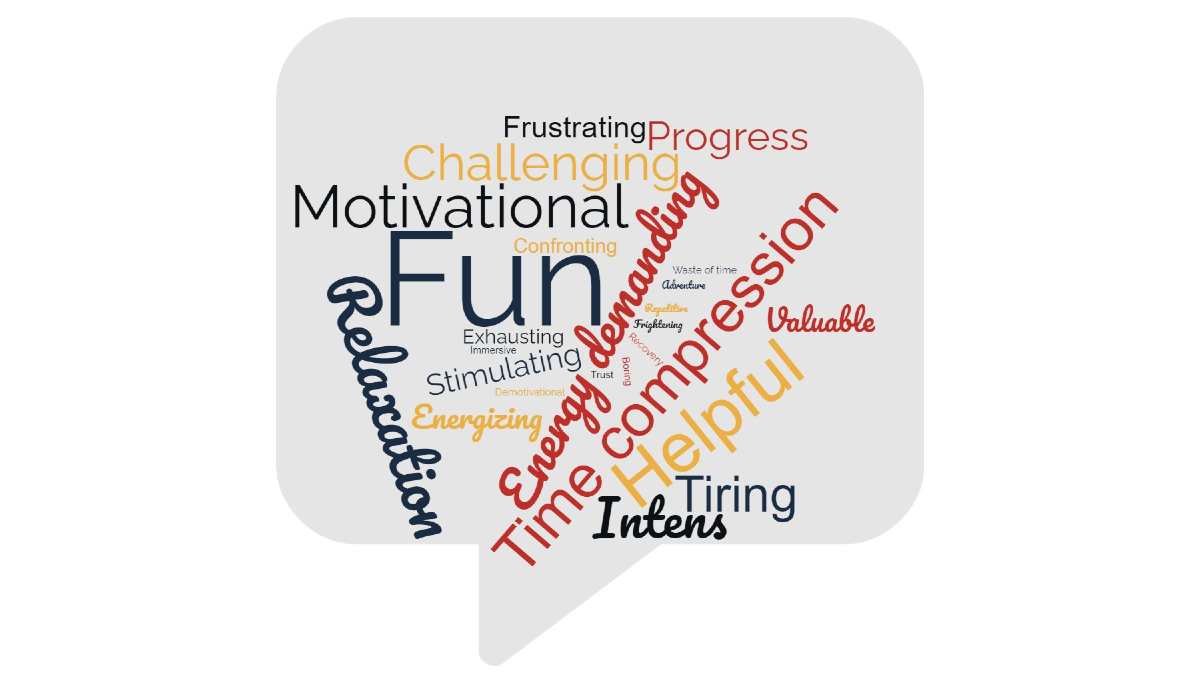
Word cloud of patients’ remarks regarding virtual reality exercising.

In the weekly telephone calls, patients used mostly positive words to describe VR, such as “fun,” “motivational,” “stimulating,” “relaxing,” “valuable,” and “energizing.” Some patients described it as “intense,” “tiring,” “confronting,” “boring,” and “energy demanding” (Figure 2). The terms “energizing” and “energy demanding” revealed a contrast between 10 patients who felt VR was too energy demanding while resuming work after sick leave and 3 patients who found the relaxation exercises energizing, especially after work. There were 3 patients who felt that VR would have benefitted them more when used early after COVID-19.

The median (range) scores of the treatment satisfaction questionnaire were 58% (33%-100%) for effectiveness, 100% (41%-100%) for side effects, 72% (33%-100%) for convenience, and 67% (33%-100%) for global satisfaction. In total, 78% (28/36) of patients would like to reuse VR in case they would need rehabilitation in the future, and 92% (33/36) of patients would recommend the VR intervention to others.

#### Usability

The median VR use frequency was 3 to 4.5 times a week for 95-115 minutes per week (Table 2). The duration of individual VR sessions varied between 5-165 minutes with a median duration of 30 minutes. There were large variations between individuals in the use of the applications in the different domains. Of the 643 sessions, cognitive exercises (n=257, 40%) were used somewhat less than physical exercises (n=344, 53.5%). However, the use frequency of cognitive exercises was more stable over time, whereas the use of physical exercises seemed to decrease over time (Table 3). Relaxation exercises were performed in 52.6% (n=338) of all sessions, and use remained relatively stable over time.

The 24/7 support line was primarily used by patients to report technical problems. In total, 40 technical problems were reported by 14 patients. Most problems related to the battery, which could be resolved by charging the head-mounted display or changing the batteries of the controllers. Some patients experienced difficulties with operating the applications. These problems could be remotely solved by the study staff. There were 3 head-mounted displays that needed to be replaced due to missing applications or a defective controller. Additionally, a software update during the intervention period caused considerable inaccuracy of digital tracking, and 7 patients were unable to use the VR intervention for 4-7 days.

**Table 2:** Frequency and duration of virtual reality use by patients who completed at least 3 weeks of virtual reality exercises.

Characteristic	Week 1 (n=34)	Week 2 (n=33)	Week 3 (n=32)	Week 4 (n=32)	Week 5 (n=31)	Week 6 (n=27)
Frequency^a^, median (IQR)	4.5 (3.0-6.0)	4.0 (2.0-6.0)	3.0 (2.0-5.0)	3.0 (2.0-5.8)	3.0 (2.0-6.0)	3.0 (2.0-5.0)
Duration^b^ (min), median (IQR)	115.0 (66.3-161.3)	90.0 (45.0-170.0)	107.5 (52.5-123.8)	95.0 (60.0-165.0)	97.5 (50.0-163.8)	95.0 (63.8-150.0)

^a^Number of sessions.

^b^Total per week.

**Table 3:** Frequency of virtual reality use by patients who completed at least 3 weeks of virtual reality exercises divided by physical, cognitive, and relaxation exercises.

Exercise		Week 1	Week 2	Week 3	Week 4	Week 5	Week 6	Overall
**Physical**								
	Patients^a^, n/N (%)	28/34 (82.4)	25/33 (75.8)	24/32 (75)	21/32 (65.6)	18/31 (58.1)	18/27 (66.7)	31/34 (91.2)
	Sessions, n/N (%)	78/643 (12.1)	65/643 (10.1)	51/643 (7.9)	58/643 (9)	51/643 (7.9)	41/643 (6.4)	344/643 (53.5)
**Cognitive**								
	Patients^a^, n/N (%)	15/34 (44.1)	19/33 (57.6)	24/32 (75)	17/32 (53.1)	16/31 (51.6)	12/27 (44.4)	28/34 (82.4)
	Sessions, n/N (%)	44/643 (6.8)	45/643 (7)	51/643 (7.9)	45/643 (7)	39/643 (6.1)	33/643 (5.1)	257/643 (40)
**Relaxation**								
	Patients^a^, n/N (%)	20/34 (58.8)	24/33 (72.7)	23/32 (71.9)	24/32 (75)	24/31 (77.4)	23/27 (85.2)	33/34 (82.4)
	Sessions, n/N (%)	56/643 (8.7)	58/643 (9)	55/643 (8.6)	54/643 (8.4)	56/643 (8.7)	59/643 (9.2)	338/643 (52.6)

^a^Number of patients that used exercises at least once in the corresponding week.

#### Tolerability and Safety

Of the 47 patients, 33 (70%) reported VR-related adverse events at least once in the diary or telephone interview. Most frequent adverse event was dizziness (n=21, 45%), followed by headache (n=10, 21%; Table 4). Notably, 25% (9/36) of the participants reported adverse events in the treatment satisfaction questionnaire, taken after the VR treatment. No falls or near falls due to VR use were reported. Additionally, 2 patients reported self-measured falls in oxygen saturation when performing physical exercises for a longer period of time (over 30 minutes); these were considered serious adverse events.

**Table 4:** Adverse events reported at least once in diary and telephone interviews.

Adverse event	Patient (N=47), n (%)
Dizziness	21 (45)
Headache	10 (21)
Fatigue	6 (13)
Nausea	7 (15)
Noncardiac chest pain	3 (6)
Neck pain	3 (6)
Blurred vision	2 (4)
Anxiety	1 (2)
Hot flashes	1 (2)
Dry eyes	1 (2)
Dyspnea	1 (2)
Restlessness	1 (2)

#### Physical Function

Significant improvements were found in the 6-MWT, grip strength, 30-CST, Borg scale on fatigue, and PSC on all 3 activities (PSC 1, 2, and 3; Table 5). There were 3 patients who performed the TUG instead of the 6-MWT, with scores between 5.0 to 17.0 seconds before and 4.2 and 9.0 seconds after the intervention. Additionally, 2 patients performed the 5-times stand test instead of the 30-CST, with before and after scores of 17.7 and 7.4 seconds and 23.9 and 13.6 seconds, respectively. Lower extremity strength was measured with a microFET dynamometer (Hoggan Scientific) in 4 participants, with before and after percentages of from 57% to 79% and from 67 to 84%, respectively. No significant changes were seen in the scores on the different domains of the NEADL.

**Table 5:** Physical function, mental function, and quality of life outcome measures before and 6 weeks after virtual reality exercises in patients who performed virtual reality exercises for at least 3 weeks. Patients with missing baseline or final measurements were excluded from analysis.

Measurement (range)		Before	After	Mean difference (95% CI)	*P* value
**Positive Health (n=36)**					
	Total score (0-420), mean (SD)	297.5 (41.0)	307.9 (43.8)	10.4 (0.7-19.9)	.04^a^
	Bodily functions (0-70), mean (SD)	38.7 (8.4)	44.2 (10.3)	5.5 (2.9-8.0)	<.001^a^
	Mental well-being (0-70), mean (SD)	45.8 (9.1)	49.4 (9.2)	3.6 (0.9-6.2)	.01^a^
	Meaningfulness (0-70), mean (SD)	50.8 (7.8)	52.0 (8.3)	1.2 (–0.6 to 3.0)	.19^a^
	Quality of life (0-70), mean (SD)	52.3 (8.0)	53.3 (7.7)	1.0 (–0.7 to 2.6)	.25^a^
	Participation (0-70), mean (SD)	57.5 (6.9)	57.0 (6.6)	–0.5 (–2.6 to 1.6)	.63^a^
	Daily functioning (0-70), median	53.5	53.0	—^b^	.78^c^
**Short Form-12 (n=36)**					
	Physical (0-100), mean (SD)	34.9 (8.3)	36.4 (9.5)	1.5 (0.01-3.08)	.049^a^
	Mental (0-100), mean (SD)	44.0 (8.8)	47.5 (9.1)	3.5 (0.76-6.07)	.01^a^
CFQ^d^ (0-100; n=36), median		37.5	31.5	—	.11^c^
**HADS^e^ (n=36)**					
	Total score (0-42), mean (SD)	11.6 (5.1)	10.2 (5.6)	–1.4 (–2.8 to 0.2)	.08^a^
	No generalized anxiety disorder (0-7), n (%)	28 (78)	29 (81)	—	—
	Possible generalized anxiety disorder (8-10), n (%)	5 (14)	6 (17)	—	—
	Likely generalized anxiety disorder (11-21), n (%)	3 (8)	1 (3)	—	—
	No major depressive episodes (0-7), n (%)	26 (72)	26 (72)	—	—
	Possible major depressive episodes (7-10), n (%)	8 (22)	7 (19)	—	—
	Likely major depressive episodes (11-21), n (%)	1 (3)	2 (6)	—	—
**NEADL^f^ (n=36)**					
	Mobility (0-18), mean (SD)	14.0 (2.8)	14.5 (2.3)	0.5 (–0.3 to 1.4)	.22^a^
	Kitchen (0-15), median	15.0	15.0	—	.25^c^
	Domestic (0-15), median	13.0	12.0	—	.86^c^
	Leisure (0-18), mean (SD)	11.8 (4.2)	12.1 (4.3)	0.3 (–1.1 to 1.6)	.71^a^
PSC^g^ 1 (0-100; n=35), mean (SD)		71.3 (20.1)	43.9 (25.4)	–27.4 (–36.5 to –18.3)	<.001^a,h^
PSC 2 (0-100; n=30), mean (SD)		64.1 (18.9)	36.8 (24.2)	–27.3 (–35.7 to –19.1)	<.001^a,h^
PSC 3 (0-100; n=24), median		80.0	50.0	—	<.001^c^
6-MWT^i^ (m; n=33), median		462.5	522.5	—	<.001^c,h^
Grip strength (kg; n=30), median		29.0	29.8	—	.01^c,h^
30-CST^j^ (repetitions; n=31), median		13.0	15.0	—	.02^c,h^
Borg fatigue scale (0-10; n=36), median		5.0	4.0	—	.03^c,h^

^a^Paired samples 2-tailed *t* test.

^b^Not available.

^c^Wilcoxon signed-rank test.

^d^CFQ: Cognitive Failure Questionnaire.

^e^HADS: Hospital Anxiety and Depression Scale.

^f^NEADL: Nottingham Extended Activities of Daily Living questionnaire.

^g^PSC: Patient-Specific Complaints questionnaire.

^h^Clinically relevant improvement (>10%).

^i^6-MWT: 6-Minute Walk Test.

^j^30-CST: 30-Second Chair Stand Test.

#### Mental Function

The scores of the Positive Health questionnaire and SF-12 were significantly increased after 6 weeks. The 1.4 point decrease of total HADS score was not significant (*P*=.08) for the total group but reached significance (*P*=.01) for the subgroup of patients who used the mental VR applications.

Cognitive failure, as measured by the CFQ, did not significantly decrease, both in the whole group and the subgroup of patients who used the cognitive exercise application Koji’s Quest.

#### Correlations Between Patient and Disease Characteristics and Functions and Quality of Life Outcomes

A positive correlation was found between the duration of VR use and age (*r*=0.57; *P*<.001). Other patient and disease characteristics did not show any significant correlation. The duration of VR use did not correlate with physical and mental functions or quality of life outcomes.

## Discussion

### Principal Findings

This study demonstrates that the use of VR for physical and self-administered mental exercising at home is feasible and appreciated in about three-quarters of patients with post–COVID-19 condition. Patients spent on average over 90 minutes per week exercising in VR. The overall use of VR applications was almost equally distributed over the 3 sets of VR exercises (physical, relaxing, and cognitive), although only physical exercises were prescribed, and considerable individual variations existed. Several physical function outcomes, perceived positive health, and quality of life improved in time, whereas cognitive function seemed unaltered. The design of the study did not allow for establishing improvement as a sole benefit of VR exercises. The results show patients’ need for mental rehabilitation in addition to physical rehabilitation and the potential of self-administered VR in the recovery from post–COVID-19 condition.

The main study aim was the feasibility of self-administered VR physical exercises at home. The design was chosen accordingly, which does not allow conclusions to be drawn on effectiveness in comparison with standard rehabilitation alone. This precaution mainly regards physical function, because one might doubt similar mental effects with standard home exercise instructions by physiotherapists. An effect of the natural course of recovery cannot be ruled out, although it is less likely considering the long duration of symptoms at study entry in most patients. This study was largely conducted before the availability of vaccination and in a time of social distancing and reluctance of physical encounters. This may have affected the appreciation of home VR exercising programs by patients and physiotherapists and the acceptability and usability results in both positive and negative directions.

This practice-based study was performed in the primary care setting and included a representative group of patients with a variety of symptoms of post–COVID-19 condition and rehabilitation needs [43,44]. Physiotherapists and patients were engaged early in the study design for defining relevant VR content, inclusion criteria, and outcome measures according to the most recent rehabilitation standards. This design benefits the generalizability of the study results. However, one could question the generalizability when taking into account the selection of therapists with a holistic approach to physiotherapy and the inclusion of patients who were capable of exercising at home with VR. This selection bias may have affected the appreciation, feasibility, and other outcomes of VR exercises, particularly in the mental domain. Conceivably, physiotherapists have emphasized the importance of stress and anxiety reduction and cognitive function along with physical recovery for restoring the health-related quality of life and participation.

One-quarter of the patients discontinued VR use before study end, in which half were due to adverse events, particularly dizziness. Dizziness is a common adverse effect of VR in general. The almost 50% of patients complaining of dizziness at some point in the 6-week treatment period seems high compared to VR in other areas, such as pain management or stress therapy [45]. This finding may be explained by concomitant complaints in the context of post–COVID-19 condition, such as fatigue, balance disturbances, and “brain fog.” Notably, only 25% of the patients recalled having experienced any adverse events at the end of the intervention period. This may imply that the symptoms were relatively mild. The dropout rate of 15% due to adverse events in this study was comparable to the mean dropout rate of 16% reported in a recent systematic review on factors associated with VR adverse events [28]. A potential factor affecting dizziness and nausea, both symptoms of “VR sickness,” is a prolonged playing time per session and possible latency in the software of physical exercising applications [28]. Although patients were instructed not to exceed 30 minutes, a considerable number did, because they lost track of time when immersed in the virtual world, particularly when performing physical exercises. Time compression is a known phenomenon in VR, which contributes to the benefit of VR in acute pain management [46]. Some participating physiotherapists initially observed serious falls in oxygen saturation levels after a few minutes of supervised VR physical exercises, which remained unnoticed by patients. This was an important reason to continue supervised sessions in the office for a few times and urge these patients not to exceed the exercise time. The 2 occasions of oxygen saturation falls reported by patients should be considered a serious adverse event of unsupervised VR physical exercising, in particular when followed by a postexertional symptom exacerbation [47]. Prolonged exercising due to time compression prompted us to instruct all patients to set an alarm when exercising alone at home.

We found a positive correlation between the duration of VR use and age. There may be several explanations for this result. Older patients may have been slower in using the applications than younger patients, because they are not used to navigating through the application menu or with the controllers. Older patients may be more immersed in the virtual environment and are more curious about this “new” experience, prolonging the time of VR use. Conversely, younger patients might become bored earlier, as they are used to gaming in VR [48]. Finally, attitude toward a prescribed therapy might differ between age groups, with older patients being more adherent than younger patients. Age differences in acceptation, usability, tolerability, and the effects of VR have been described in numerous papers, however, with equivocal results. Our feasibility study, with a limited group of patients, did not allow for the analysis of other individual characteristics related to VR use. From the results of the digital health design evaluation study, we demonstrated a complex interplay between patients’ beliefs and values about VR use, such as autonomy, social comfort, self-identity, privacy, and its effects on recovering from post–COVID-19 condition [26].

Between 0% to 10% of the data were missing regarding the primary outcome measures of acceptability and tolerability. However, the diaries’ data were increasingly missed over time in 15% to 32% of the patients, most likely because they forgot to answer the same questions daily, which would mean that the actual use was higher than the reported use of VR. Automated collection of data from the headset would have benefitted the accurate assessment of the type, level, frequency, and duration of VR use. However, this functionality was not made available in the software (eg, application type and level) or was hampered by technical problems (eg, Wi-Fi connection and interim updates). Function outcomes were missing in 10% to 25% of patients, possibly due to delayed evaluations and administration faults by the physiotherapists. Accordingly, we cannot rule out an overestimation of these outcomes in this study.

### Comparison to Prior Work

The frequent use of mental exercise applications at the patients’ own initiative in this study underlines the needs of patients with post–COVID-19 condition for a multimodal rehabilitation approach. However, most patients were only referred for physiotherapy as a single treatment. This finding reflects the emphasis on the physical domain of rehabilitation in most studies, although guidelines include multidisciplinary rehabilitation after COVID-19 for both hospitalized and nonhospitalized patients [43,49]. Daynes et al [50] evaluated a multimodal, home-administered, post–COVID-19 rehabilitation program for feasibility; however, they did not evaluate VR. The study duration was 6 weeks with 2 supervised sessions per week. The sessions comprised of physical exercises and educationally oriented conversations regarding mental complaints. The authors reported improvements in physical and cognitive functions but not regarding anxiety and depression. A recent study exploring the feasibility of VR relaxation games found similar results to our study with high patient satisfaction and benefits regarding mental function; however, this study concerned inpatient post–COVID-19 rehabilitation [25]. Multimodal VR has been used in poststroke rehabilitation, resulting in improved physical and cognitive functions [21,51]. The differences in domains regarding effect might be due to the design of the VR intervention. Purpose-designed VR interventions seem to be more effective [52]. When we selected the applications, little was known about post–COVID-19 condition, and therefore, we chose a broad range of existing applications.

### Strengths and Limitations

The strength of this study is the comprehensive collection of feasibility and function data using a novel approach of rehabilitation for post–COVID-19 condition with VR exercises. The study was designed to allow patients a lot of autonomy in choosing exercises in both the physical and mental domains, reflecting the real needs and wishes of the patients with this condition.

This study also has limitations. First, the approach was mono-professional for a postinfectious condition with symptoms that commonly require a multiprofessional approach. Accordingly, VR exercises to improve cognitive function and reduce stress and anxiety were not prescribed in contrast to physical exercises. However, by selecting physiotherapists with a psychosomatic approach and experience in treating similar postinfectious conditions, attention given to mental recovery might have been higher compared to the average physiotherapists’ approach. A further limitation in this context is the absence of information regarding concurrent treatment by an occupational therapist and a psychologist, which may have affected mental function outcomes and the quality of life. The mono-professional approach likely made no difference for the feasibility outcomes of VR exercises. Second, the study was conducted in the first year of the pandemic with limited knowledge of the cause of symptoms and scarce evidence on post–COVID-19 rehabilitation. Despite the reference to follow the actual guidelines in the protocol, this setting may have resulted in a multiformity of physiotherapy approaches, such as different uses of physical metrics and performance measurements, different indications for self-administered VR exercises at home, and different follow-up schemes. Notably, the guidelines considered standard and not VR physical exercises. Third, the first use of the multimodal VR suite for this condition and for use at home came with several organizational, technical, and monitoring challenges. We attempted to mitigate these challenges by supporting physiotherapists and patients through distributing, disinfecting, and administrating the hardware, providing around-the-clock (technical) support and performing weekly telephone calls. Against the background of limited physiotherapy resources and social distancing, this support may have positively affected feasibility outcomes despite the strict contact protocols with the patients and physiotherapists by the research team. This support by a research team does not reflect the normal practice of physiotherapy, which reduces the relevance of the results.

### Future Directions

The results of this study show that a self-administered multimodal VR intervention at home is feasible and safe. Many rehabilitation programs for other conditions are based on the same needs, implying that VR might benefit rehabilitation in general. The deployment of VR in rehabilitation could be administered to patients with the means to recover at home; thus, patients would not have to travel to a physiotherapist or clinic multiple times a week. Physiotherapists would be able to monitor patients’ progress at a distance to relieve workload. Telerehabilitation, including wearables for (vital sign) monitoring, analytic platforms with patient and provider dashboards, and video consulting equipment, is increasingly available for use to administer virtual physiotherapy [53,54]. It can be expected that over time VR technology will become more affordable and more easily accessible [55]. This trend might eventually result in a reduction of health care costs.

Regarding the ongoing pandemic, an increase of patients with post–COVID-19 condition is expected to increase demand for rehabilitation. Self-administered VR rehabilitation at home for physical and mental impairments can be a novel means to restore the functional status and well-being of patients with post–COVID-19 condition that is inclusive, adopted by caregivers, and sustainable and moves care from hospital and practices to at home. A wide range of VR applications in different domains motivates patients to exercise, improving therapy adherence and self-efficacy. Remotely monitoring adherence and progress in recovery and accordingly adapting the treatment plan can be a safe alternative to routine, unsupervised home exercising and regular patient visits to the physiotherapists’ office. Before broad implementation, it is recommended to perform controlled trials on the cost-effectiveness of VR for the rehabilitation of post–COVID-19 condition. The results of this study have provided several substantive and organizational leads to future research delineating the health, societal, and economic impact of VR use in the rehabilitation of post–COVID-19 condition.

### Conclusion

We found that 6 weeks of VR physical and mental exercises at home is feasible, well accepted, and safe in patients with post–COVID-19 condition, with improvements in physical and mental functions and the health-related quality of life.

## Appendix

Multimedia Appendix 1 Red flags.

Multimedia Appendix 2 Oculus Quest instructions for patients.

## Abbreviations

30-CST: 30-Second Chair to Stand Test
6-MWT: 6-Minute Walk Test
CFQ: Cognitive Failure Questionnaire
HADS: Hospital Anxiety and Depression Score
NEADL: Nottingham Extended Activities of Daily Living score
PSC: Patient-Specific Complaints questionnaire
SF-12: Short Form-12
TUG: Timed Up and Go Test
VR: virtual reality

## Data Availability

The data sets generated during and/or analyzed during the current study are available from the corresponding author on reasonable request.
