# Supplementary material for: Feasibility of Virtual Reality Exercises at Home for Post–COVID-19 Condition: Cohort Study
Source: JMIR Rehabil Assist Technol. 2022 Aug 15;9(3):e36836. doi: 10.2196/36836 (PMC9380776; doi:10.2196/36836)
Supplement: Multimedia Appendix 1 [file rehab_v9i3e36836_app1.docx]

**Appendix 1. Red flags for physical exercises according to guidelines.***

Stop examination/treatment and consult a physician in case of:

- Resting pulse <40 or > 130 beats/minute
- Respiratory rate >40 /minute
- Saturation in rest < 90% and/or during exercise <85%
- Cardiac arrythmias
- Deep vein thrombosis (DVT)
- Acute dyspnoea (with suspected lung embolism)
- Recent myocardial ischemia
- Excessive transpiration, different complexion , fear

Stop examination/treatment and repeat at other moment in case of:

- Fever (body temperature >38,0 degrees Celsius
- Exhaustion (Borg scale ≥5 in rest)
- High blood pressure in rest (≥180/100)

* Recommendations for physiotherapy in patients with COVID-19 [2.0]. Amersfoort, The Netherlands: Royal Dutch Society for Physical Therapy (KNGF); 2020.
