# Supplementary material for: Feasibility of Virtual Reality Exercises at Home for Post–COVID-19 Condition: Cohort Study
Source: JMIR Rehabil Assist Technol. 2022 Aug 15;9(3):e36836. doi: 10.2196/36836 (PMC9380776; doi:10.2196/36836)
Supplement: Multimedia Appendix 2 [file rehab_v9i3e36836_app2.pdf]

Appendix 2. Patient catalogus Oculus Quest

Catalogus Oculus Quest

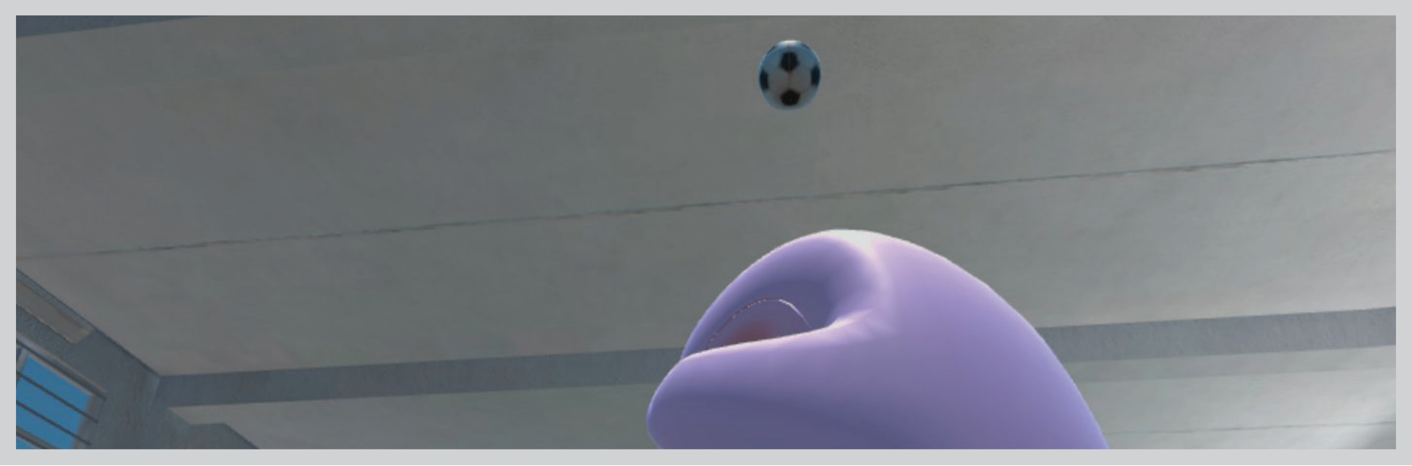

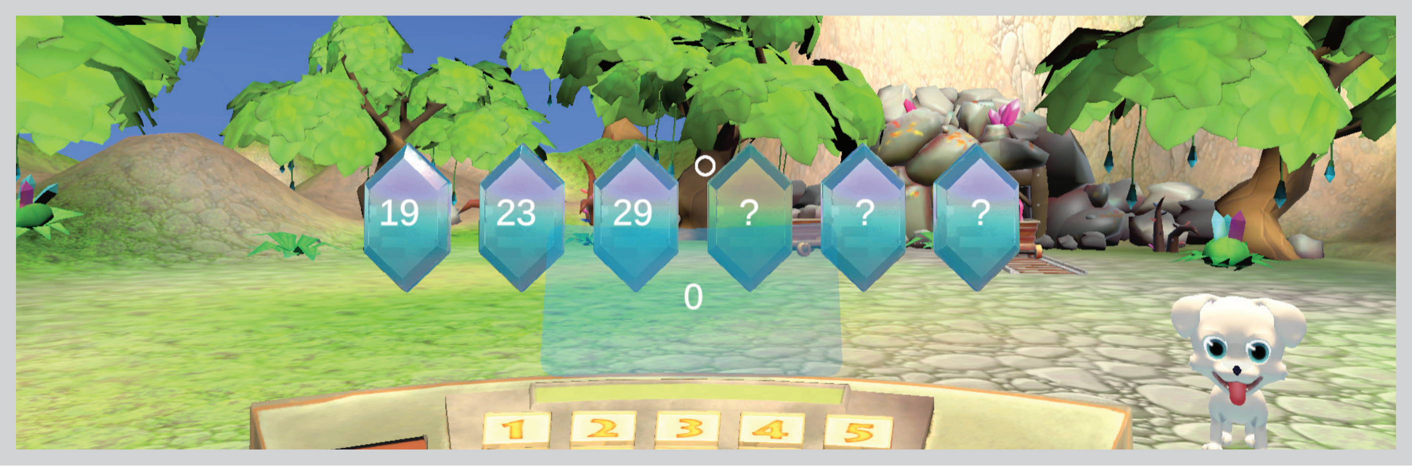

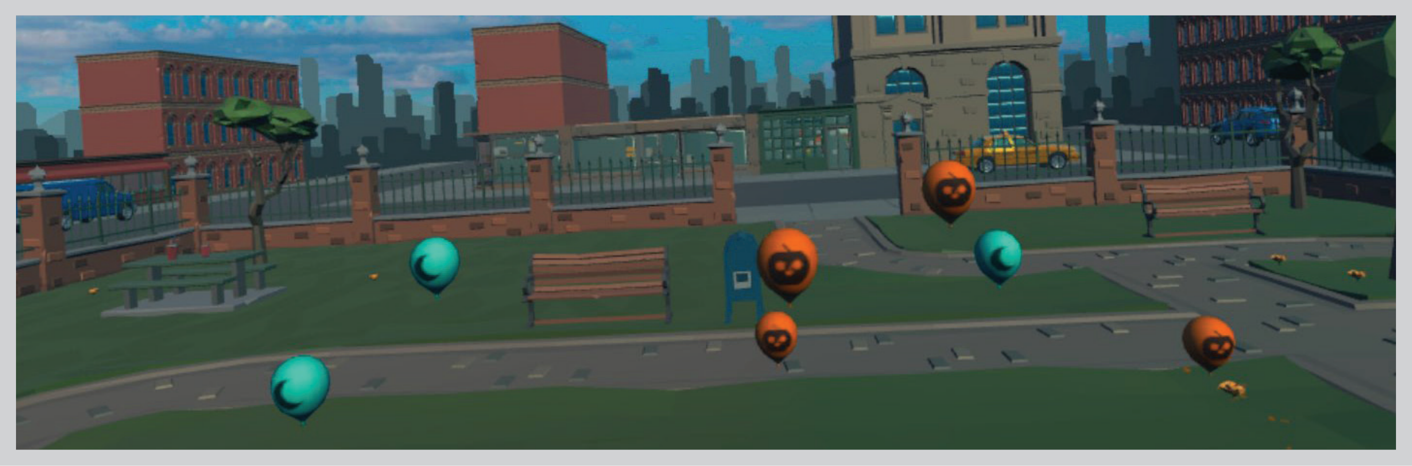

SyncVR

Catalogus

Oculus Quest

Welkom! Deze catalogus legt u in detail uit welke drie typen spellen u kunt spelen in de Virtual Reality bril. Zo kunt u samen met uw fysiotherapeut besluiten welke spellen het best passen bij uw revalidatie.

Probeer om de Virtual Reality bril te gebruiken volgens instructies van uw fysiotherapeut. Vergeet niet om elke dag dat u de Virtual Reality bril gebruikt kort het dagboekje bij te houden.

Raadpleeg de handleiding van de Oculus Quest voor het opstarten van de diverse applicaties.

Heeft u vragen? Vraag dan een van de onderzoekers om hulp.

Mail: Linda.garms@radboudumc.nl of Merlijn.smits@radboudumc.nl
Tel: 06 557 40 313

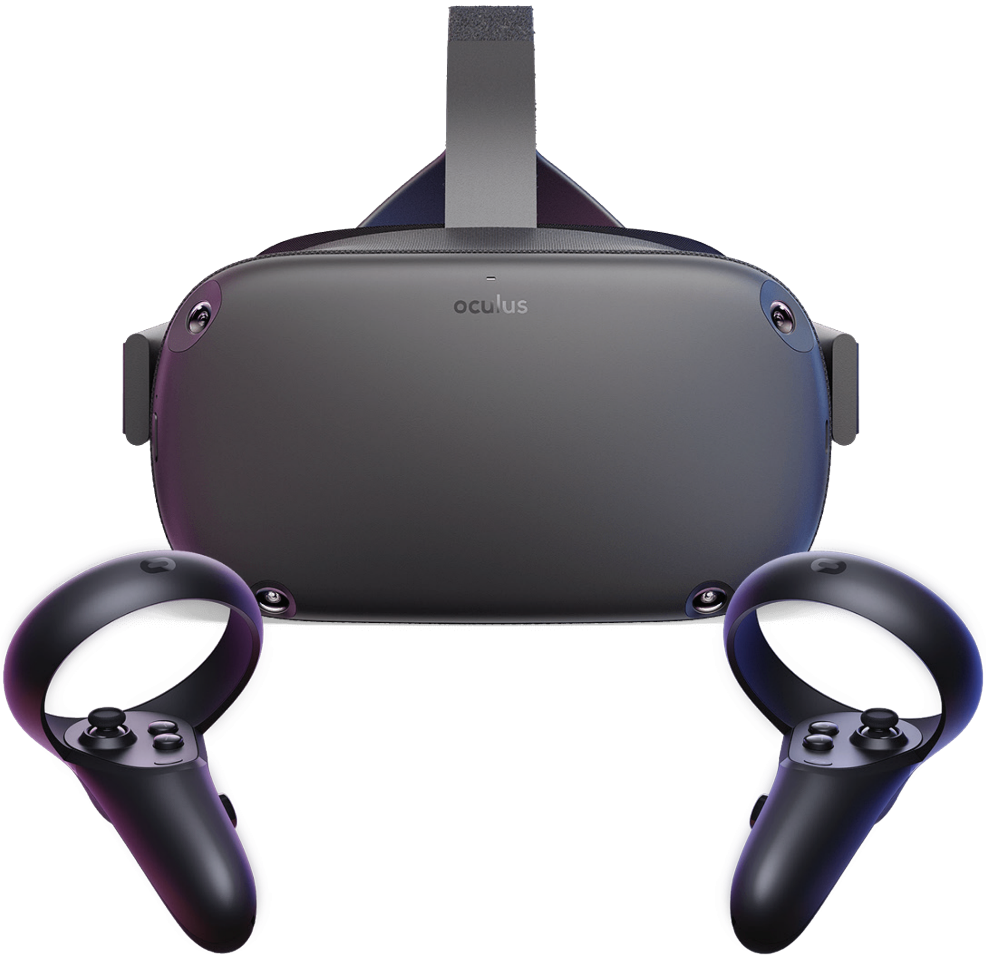

Virtual Reality revalidatie

Revalideren met Virtual Reality?

Virtual Reality kan een bijdrage leveren aan mensen die revalideren na COVID-19. Revalidatie na zo'n periode is een intensief proces waarbij, afhankelijk van de persoon, verschillende elementen aandacht vereisen. Zo is het belangrijk dat er aandacht wordt besteed aan de fysieke revalidatie om spierkracht te herstellen. Daarnaast is het ook belangrijk dat iemand de tijd krijgt om de heftige periode te verwerken en voldoende ontspanning kan vinden. Ook kan er aandacht nodig zijn om concentratie en geheugen na de ziekte te verbeteren.

In deze studie bieden we spellen aan met betrekking tot deze drie gebieden:

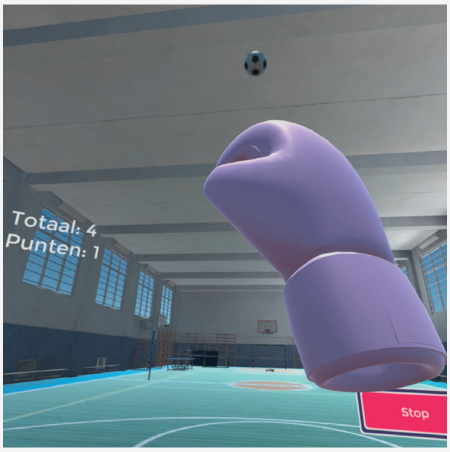

1: Bewegingsoefeningen

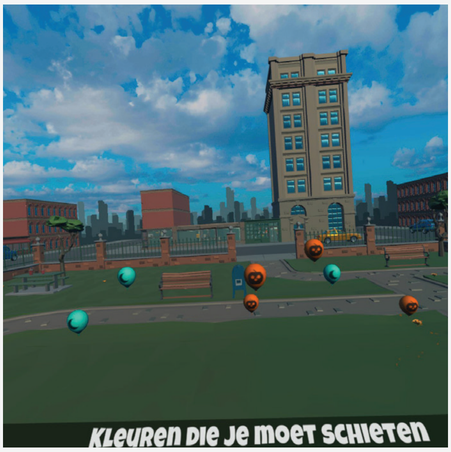

2: Ontspanningsoefeningen

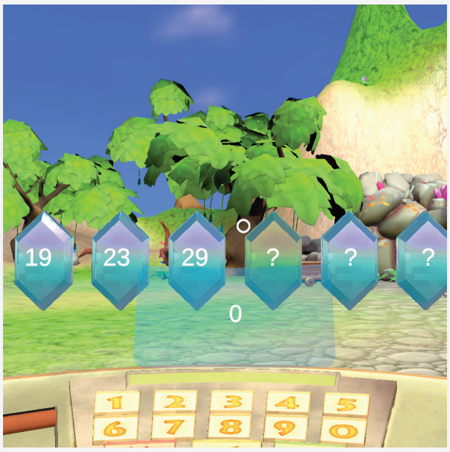

3: Cognitieve training

Deze catalogus legt per gebied (beweging, ontspanning, cognitief) uit welke spellen er worden aangeboden. Zo kunt u samen met uw fysiotherapeut besluiten wat voor u het meest geschikt is.

Bewegingsoefeningen

Doel van de bewegingsoefeningen

De bewegingsoefeningen zijn erop gericht om u op een leuke manier te begeleiden in uw beweging. Ze zijn ontwikkeld door het bedrijf *SyncVR*. De oefeningen kunnen een aanvulling zijn op uw fysiotherapie.

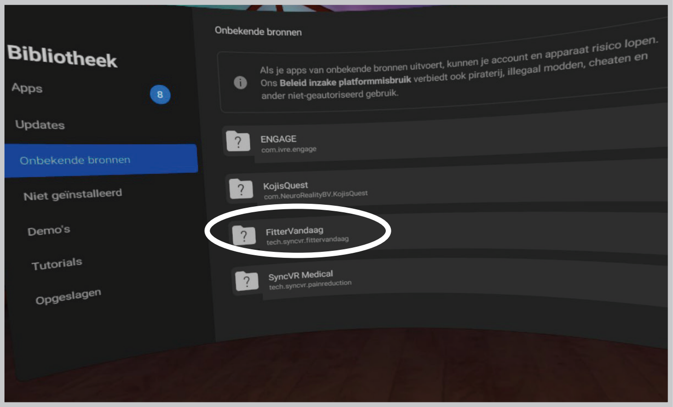

Hoe opent u de oefeningen?

Navigeer in het Oculus Home menu naar *Bibliotheek*. Klik daarna op *Onbekende Bronnen*.

Nu komt u in het menu met de verschillende spellen. Klik voor de bewegingsoefeningen op *Fittervandaag*. Het beeld blijft even zwart voordat het spel start.

Raadpleeg de handleiding voor meer informatie.

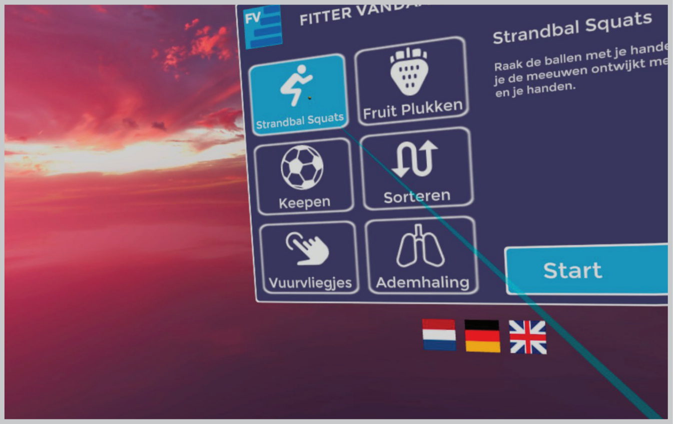

Navigatie in het spel

U komt in het hoofdmenu van *Fittervandaag*. In dit hoofdmenu kunt zes typen spellen kiezen (zie volgende pagina). Selecteer het spel naar keuze en druk op *Start*.

Het spel opent nu en legt u uit wat u moet doen.

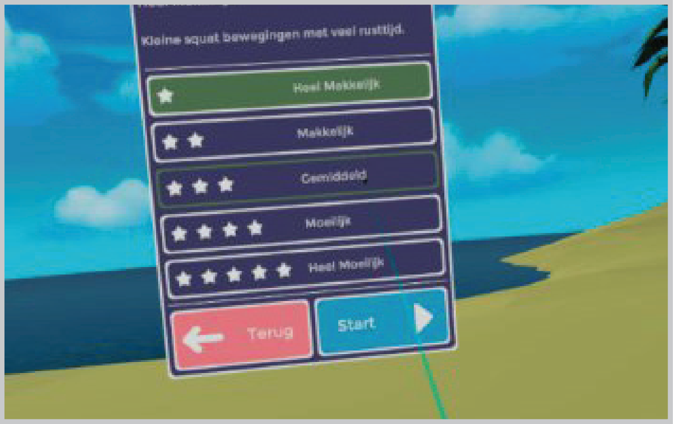

Moeilijkheidsgraad

Elk spel biedt een aantal moeilijkheidsgraden aan. Dit varieert tussen: **heel makkelijk**, **makkelijk**, **gemiddeld**, **moeilijk** of **heel moeilijk**. Bepaal met uw fysiotherapeut per spel welk niveau het best bij u past. Op den duur kunt u dan misschien een niveau hoger proberen.

Bewegingsoefeningen - spelletjes

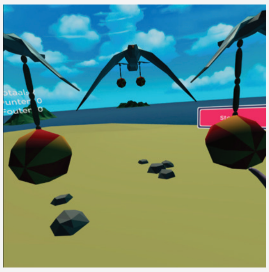

• Strandbal Squats:

Raak de strandballen met uw handen aan terwijl u de meeuwen tegelijk ontwijkt. Bij deze oefening traint u voornamelijk uw beenspieren door squats te maken. Kies uit een van de vijf niveaus het niveau dat het best bij u past. Op den duur kunt u misschien een niveau hoger kiezen.

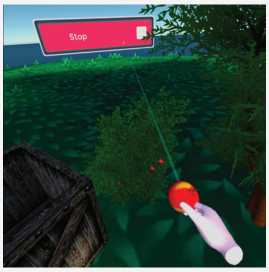

• Fruit Plukken

Pluk de bananen, appels en aardbeien uit de bomen en stop ze in het krat. Bij deze oefening traint u uw coördinatie en spieren. Er is één niveau.

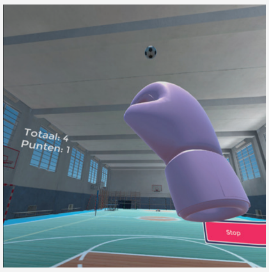

• Keepen

Houd de ballen tegen door ze weg te slaan voor ze het doel bereiken. Bij deze oefening traint u uw coördinatie en spieren. Kies uit een van de drie niveaus het niveau dat het best bij u past. Op den duur kunt u misschien een niveau hoger kiezen.

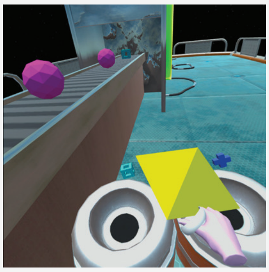

• Sorteren

Stop de objecten die over de lopende band komen in de juiste tonnen. Bij deze oefening traint u uw coördinatie, snelheid en spieren. Kies uit een van de vier niveaus het niveau dat het best bij u past. Op den duur kunt u misschien een niveau hoger kiezen.

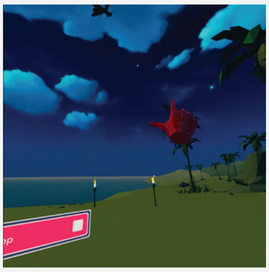

• Vuurvliegjes

Raak de vuurvliegjes aan voordat ze wegvliegen. Bij deze oefening traint u uw coördinatie en spieren. Kies uit een van de vijf niveaus het niveau dat het best bij u past. Op den duur kunt u misschien een niveau hoger kiezen.

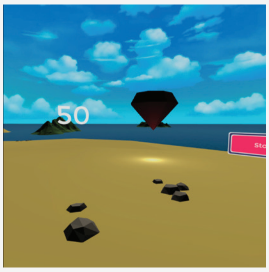

• Ademhaling

Probeer een van de drie ademhalingsoefeningen: rustige ademhaling, diepere ademhaling, huffen. Het spel legt u precies uit hoe het werkt. Bij deze oefening traint u de ademhalingsspieren en kunt u ontspannen.

Ontspanningsoefeningen

Doel van de ontspanningsoefeningen

De ontspanningsoefeningen zijn erop gericht om bij te dragen aan uw ontspanning en rust te vinden. Ze zijn ontwikkeld door het bedrijf *SyncVR*. Ze kunnen u op elk moment van de dag een momentje voor uzelf geven.

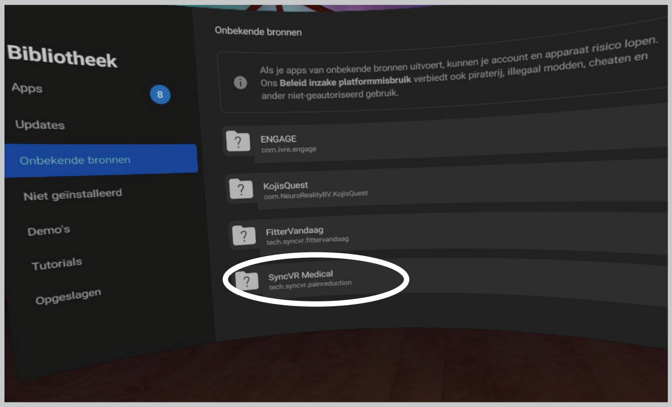

Hoe opent u de oefeningen?

Navigeer in het Oculus Home menu naar *Bibliotheek*. Klik daarna op *Onbekende Bronnen*.

Nu komt u in het menu met de verschillende spellen. Klik voor de ontspanningsoefeningen op *SyncVR Medical*. Het beeld blijft even zwart voordat het spel start.

Raadpleeg de handleiding voor meer informatie.

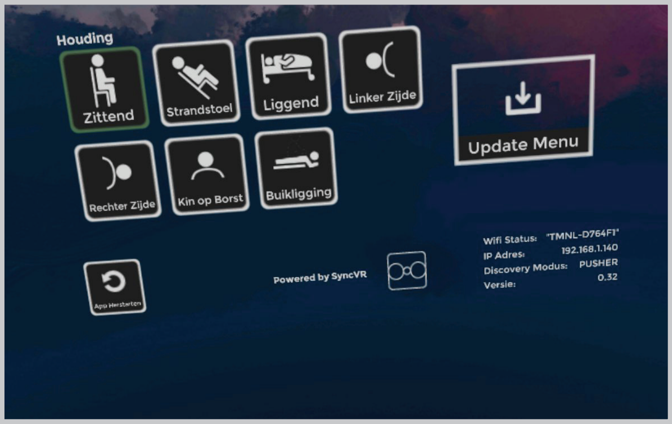

Kies uw houding

U komt in het hoofdmenu van *SyncVR Medical*. Standaard staat dit menu ingesteld op basis van een zittende houding. Wilt u de ontspanningsoefeningen liever liggend uitvoeren? Kijk dan 180 graden achter u en kies voor een andere houding. U kunt kiezen voor zitend, strandstoel, liggend, linker zijde, rechter zijde, kin op borst, buikligging.

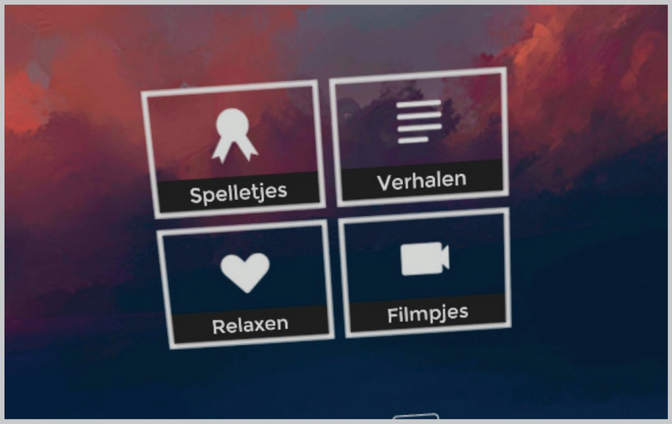

Navigatie

Na het bepalen van uw houding, kiest u uit een van de volgende opties: *spelletjes*, *verhalen*, *relaxen* of *filmpjes*.

Ontspanningsoefeningen

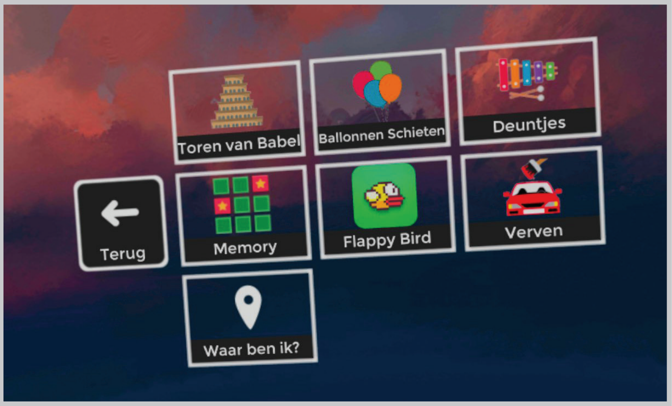

Spelletjes

Kies een van de ontspannende spelletjes uit om te spelen. Veel plezier! Een uitleg over de spelletjes kunt u op de volgende pagina lezen.

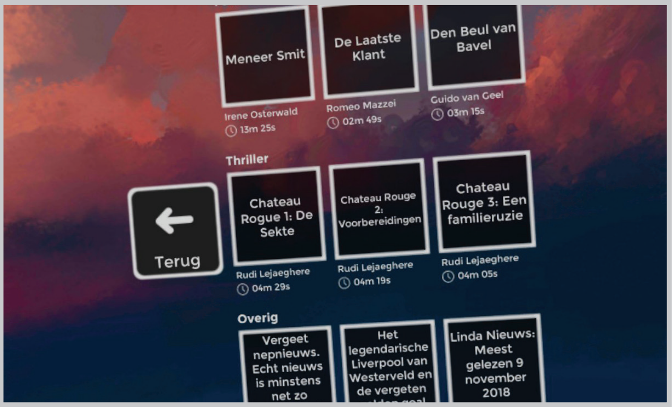

Verhalen

U kunt kiezen uit een reeks ontspannende verhalen en nieuwsitems. De woorden verschijnen een voor een in beeld. U kunt zelf de snelheid bepalen.

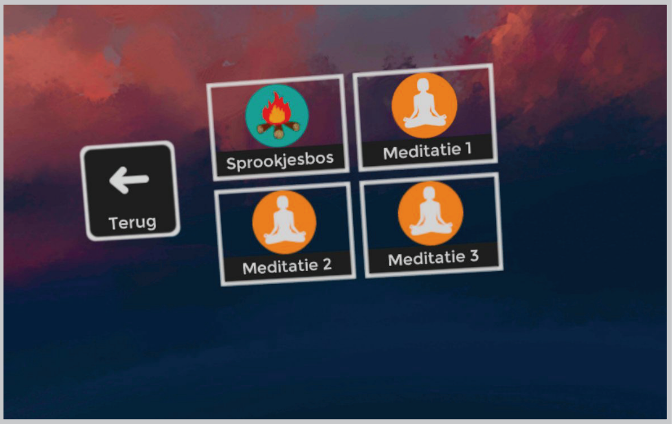

Relaxen

Kies een van de vier ontspannende meditatie oefeningen. Een rustige stem begeleidt u in een natuurlijke omgeving.

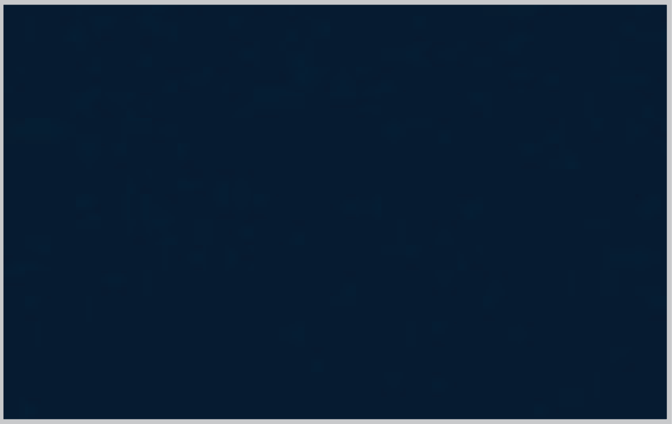

Filmpjes

Momenteel wordt hier nog niets aangeboden, hier wordt aan gewerkt.

Ontspanningsoefeningen - spelletjes

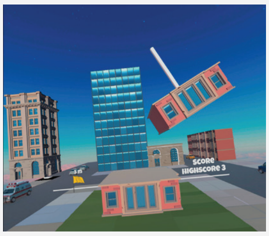

• Toren van Babel:

Bouw een zo hoog mogelijke toren van Babel. Laat het stuk telkens vallen op het juiste moment met de A-knop.

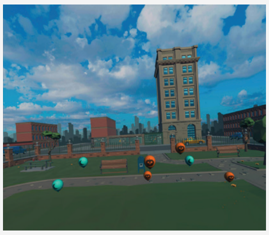

• Ballonnen schieten

Schiet de ballonnen in de juiste kleur neer. Wees op tijd, anders klappen de ballonnen. Je navigeert met de selecteerknop.

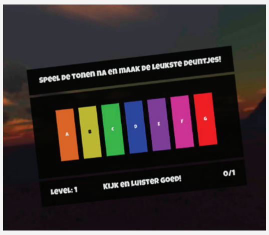

• Deuntjes

Speel de noten na, of verzin een eigen deuntje. Je navigeert met de selecteerknop.

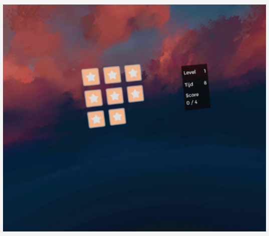

• Memory

Speel een ouderwets spelletje memorie. Hoe goed is jouw geheugen nog? Je navigeert met de selecteerknop.

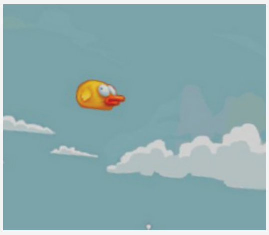

• Flappy Bird

Zorg ervoor dat het vogeltje blijft vliegen en vlieg niet tegen de muren aan. Je vliegt door de selecteerknop telkens opnieuw in te drukken.

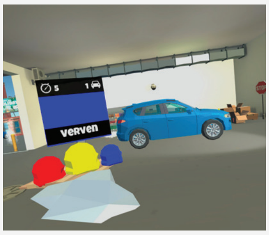

• Verven

Verf de auto in de juiste kleuren. U navigeert met de selecteerknop. Klik op de knop *Verven* zodra u de juiste kleur heeft geselecteerd.

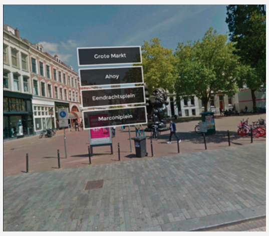

• Waar ben ik?

Je komt terecht op een bekende plaats in Nederland. Kijk goed om je heen waar je bent en selecteer het juiste antwoord uit de vier opties die je krijgt.

Cognitieve training

Doel van de cognitieve training

Koji's Quest is ontwikkeld door het bedrijf *NeuroReality* met het doel om cognitieve vaardigheden te trainen. De cognitieve vaardigheden waar Koji's Quest zich momenteel op richt zijn **Aandacht**, **Geheugen**, **Executief Functioneren**, **Visuospatiaal Redeneren** en **Rekenen**. De applicatie is dus geschikt voor personen die problemen ondervinden in deze cognitieve functies. Door deze vaardigheden te trainen met brain training games stimuleren we neuroplasticiteit; de mogelijkheden van de hersenen om nieuwe verbindingen te maken door het repetitief oefenen van taken. Koji's Quest is in beginsel ontworpen om de cognitieve vaardigheden te trainen van mensen met hersenletsel, zoals een beroerte. Omdat het duidelijk is geworden dat veel mensen na corona en na ziekenhuisopname ook cognitieve klachten ondervinden, kan Koji's Quest ook voor hun meerwaarde leveren.

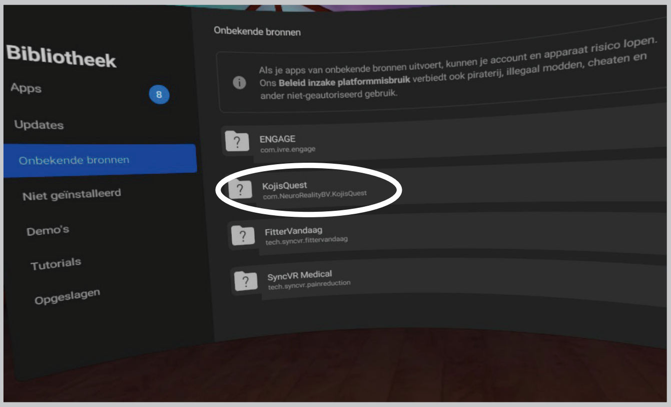

Hoe opent u de oefeningen?

Let op: zorg voor u begint dat uw VR-bril met uw wifi netwerk thuis is verbonden. Hoe u dit doet, leest u in de handleiding.

Navigeer in het Oculus Home menu naar *Bibliotheek*. Klik daarna op *Onbekende Bronnen*. Nu komt u in het menu met de verschillende spellen. Klik voor de geheugentraining op *KojisQuest*.

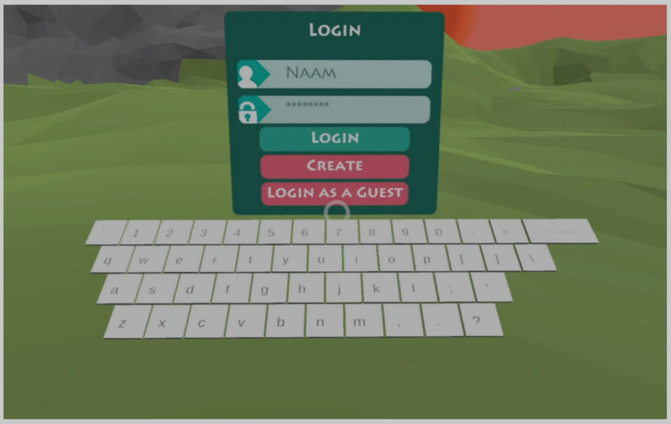

Login

De eerste keer dat u het spel opent, kunt u een gebruikersnaam en wachtwoord instellen door deze in te toetsen op het virtuele keyboard. Wanneer u vervolgens het spel afsluit, onthoud deze uw gebruikersnaam en kunt u bij de volgende keer spelen weer verder bij waar u was gebleven.

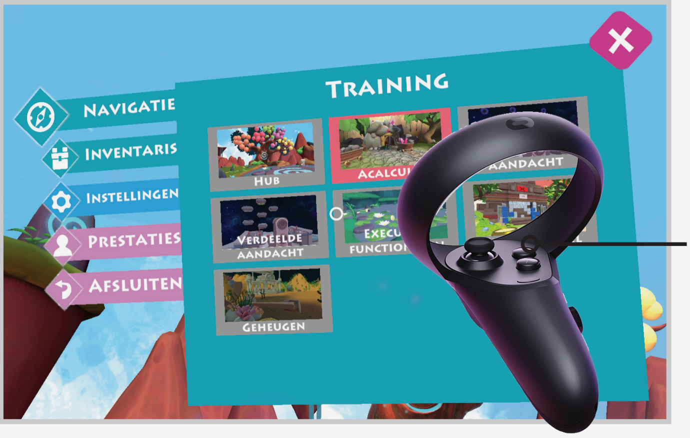

Hoofdmenu

U komt na het inloggen in de Hub omgeving. Hier kunt u aangeven hoe u zich vandaag voelt. Dit is ook het centrale punt vanuit waar u trainingen selecteert of instellingen (zoals taal en volume) aanpast.

Bent u in een spel en wilt u stoppen? Dit kan door met de controller de **B-knop** in te drukken. Het menu komt nu op. *Om het menu te deactiveren, klikt u nogmaals op de B-knop.*

Cognitieve training

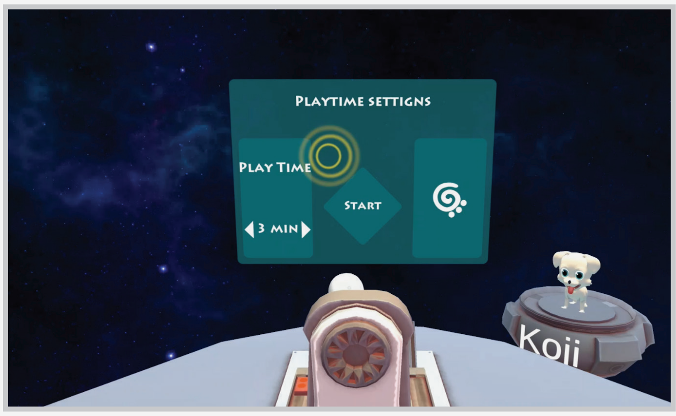

Speeltijd

U kunt zelf instellen hoe lang een training duurt. Het wordt aangeraden om de eerste keer dat u een training doet, de tijd in te stellen tussen 6 tot 10 minuten. Als u daarna korter wilt trainen per spelwereld, kunt u de tijd zelf naar beneden aanpassen. We raden aan om minimaal 30 minuten tot maximaal 60 minuten per dag Koji's Quest te spelen.

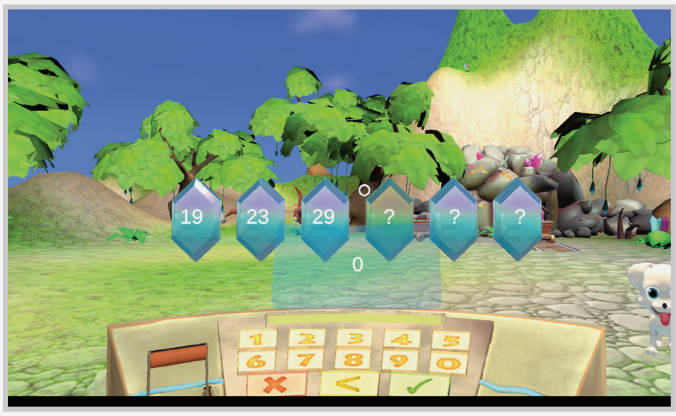

Moeilijkheidsgraad

De allereerste keer dat u een nieuwe spelwereld betreed zal de computer het moeilijkheidsniveau aanpassen op basis van uw spel. Het is van belang dat u tijdens deze spelsessie zo goed mogelijk uw best doet, en de training tussen de 6 en 10 minuten instelt, om op het juiste niveau te worden ingeschaald.

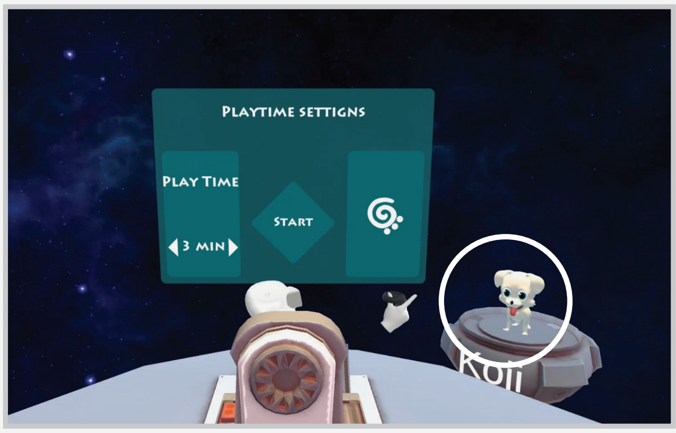

Uitleg spellen

Elk spel wordt aan het begin kort uitgelegd aan de hand van stapjes. Indien niet duidelijk genoeg, kunt u Koji, de hulphond selecteren, die vervolgens het spel eenmaal uitlegt aan de hand van kleinere stapjes. Hierna kunt u zelf aan de slag.

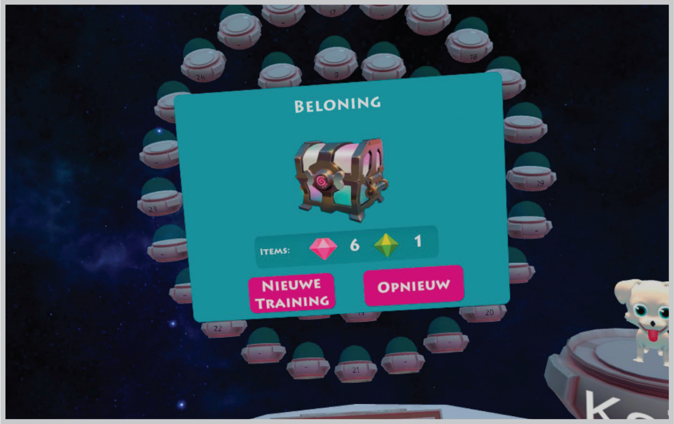

Einde spel

Wanneer de tijd voorbij is, zal de training stoppen. U krijgt nu te zien hoe goed u heeft gespeeld. Daarna kunt u een nieuwe training doen door de knop *Nieuwe Training* te selecteren, of u kunt dezelfde training nogmaals spelen, door de knop *Opnieuw* te selecteren.

Cognitieve training - spelletjes

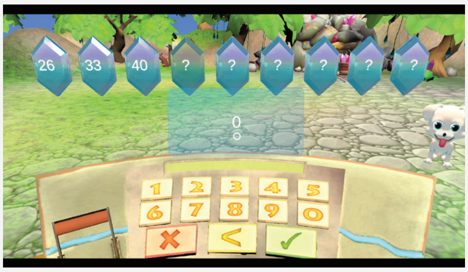

• Acalculia

In deze bosrijke omgeving train je je hersenen met diverse rekensommen. Maak telkens de getallenreeks af door de juiste getallen in te toetsen.

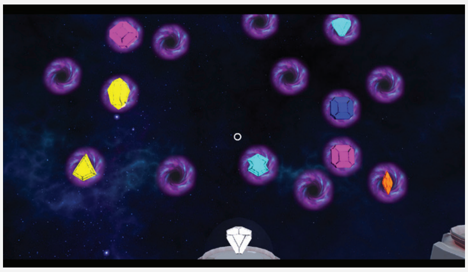

• Selectieve aandacht

In deze oefening train je je geheugen en snelheid door te zoeken naar gelijke kleur, vorm en kleur en vorm. Doe de oefening diverse malen.

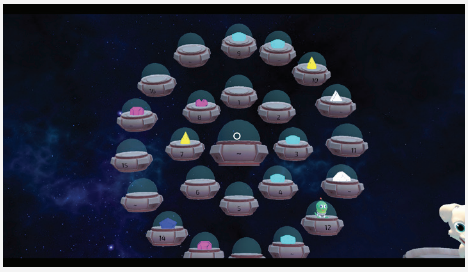

• Verdeelde aandacht

Focus op het ruimteschip voor je. Kijk naar de objecten in het ruimteschip en vergelijk ze met de objecten die buiten het ruimteschip worden aangeboden. Wees alert want de objecten zijn zo weg. De oefening wordt telkens wat moeilijker.

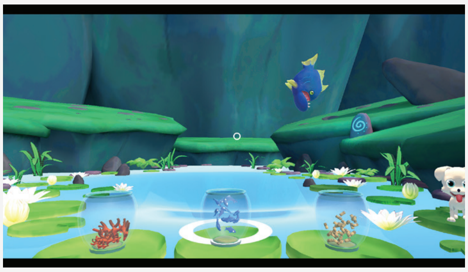

• Executief functioneren

Dit spel focust op flexibiliteit en dwingt je snel beslissingen te maken. Vissen hebben honger en moet je voeren met het juiste type voedsel. Het spel wordt steeds ingewikkelder naarmate de tijd vordert.

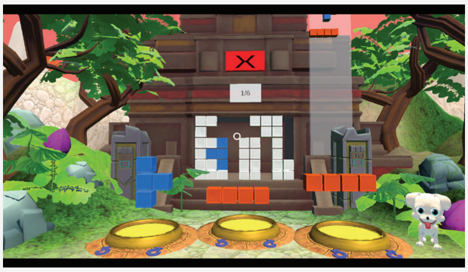

• Visuospatieel

Voor je zie je een aantal tetris blokjes. Deze moet je zodanig roteren dat ze in het gat in de muur passen. Het spel wordt steeds een beetje moeilijker.

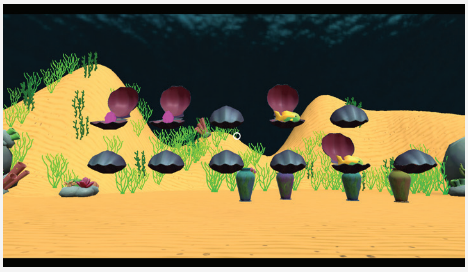

• Geheugen

In deze onderwaterwereld train je je lange en korte termijn geheugen. Je moet een aantal objecten onthouden voor kortere en langere periode en vervolgens reproduceren.
